# “We are the best to stand in for patients”: a qualitative study on nurses’ advocacy characteristics in Ghana

**DOI:** 10.1186/s12912-017-0259-6

**Published:** 2017-11-14

**Authors:** Grace Dadzie, Lydia Aziato, Ama de-Graft Aikins

**Affiliations:** 1 Department of Adult Health, School of Nursing, College of Health Sciences, University of Ghana, P. O. Box LG 43, Legon, Accra, Ghana; 2 Regional Institute for Population Studies, University of Ghana, P. O. Box LG 96, Legon, Accra, Ghana

**Keywords:** Advocacy, Characteristics, Nurse, Patient, States, Traits

## Abstract

**Background:**

Patient advocacy has been identified as a core duty of the nurse, and certain nurse characteristics influence the performance of the role. However, these characteristics have not been adequately explored in Ghana. This study aimed to explore the perspectives of nurses about the characteristics of nurses that influence their role as patient advocates.

**Methods:**

An exploratory descriptive qualitative study was conducted among 15 nurses from a regional hospital in Ghana. Purposive sampling was used to select participants and individual in-depth interviews were conducted in English using a semi-structured interview guide. The interviews were audio-taped and transcribed. Data analysis was done concurrently employing the principles of thematic analysis. Ethical approval was obtained for the study from the Noguchi Memorial Institute of Medical Research and the Ghana Health Service Ethical Review Committee.

**Results:**

Themes generated revealed nurse traits which enhanced the advocacy role of nurses such as being empathetic, nurturing, ethical, assertive and persistent and nurse states which hindered the performance of the role such as fatigue and frustration. However, “compassionate” emerged as an additional nurse trait from this study. Out of empathy, participants availed themselves for patients to share their problems with them. In their nurturing roles, spending more time with patients and providing personal care fostered closeness which helped in identifying patients’ problems. Helping patients navigate the health system was also found. They perceived patient advocacy as a moral responsibility and identified good communication skills and determination to help patients get their problems solved as important in patient advocacy. Some participants also described compassion-based activities such as pleading on patients’ behalf, providing material and financial assistance, facilitating care and providing emotional support in their advocacy. However, heavy workload and lack of appreciation from patients were found to hinder the performance of the advocacy role.

**Conclusions:**

We concluded that nurse characteristics that influence patient advocacy are comparable to those identified internationally such as being empathetic, assertiveness and fatigue. Enhancing these characteristics could help nurses overcome the negative states that undermine the patient advocacy role of nurses.

## Background

**Fig. 1:**
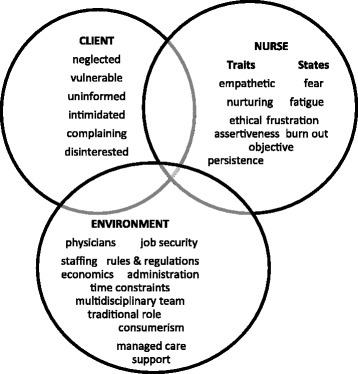
Conceptual Model of Advocacy Categories and Interaction Arenas [6]

Patient advocacy, also referred to as nursing advocacy, is the ethical practice of representing patient and family needs and wishes to ensure that medical decision making is in line with the client’s wishes [1]. Patient advocacy is a core duty of nurses [2] and nurses’ character traits such as moral courage, empathy have been found to influence the role [3–5]. The conceptual model “advocacy categories and interaction arenas” [6] among other things describes the nurse characteristics that influence patient advocacy (Fig. 1).

The model was developed to address the paucity of literature describing patient advocacy from the perspectives of practicing nurses and administrators. The authors hoped to provide a model that was grounded in both theory and research on patient advocacy to reconcile advocacy literature with different practice perspectives. The model provides clarity about how nurses view their advocacy role and the circumstances under which they exercise the role. This they believed would help nurse educators and managers enhance the practice of advocacy among students and professionals. The model emerged from a qualitative descriptive study involving 17 nurses in the USA to determine how nurses define and characterize the concept of patient advocacy. It delineates the essential components and processes of patient advocacy and provides clarity about how nurses view the role [6]. Three interdependent categories that help to determine when, if and how nurses practiced patient advocacy were identified as “client”, “nurse” and “environment”. The client exhibits characteristics that stimulate the nurse to advocate for him or her. The nurse characteristics are described in terms of traits and states. Nurse traits enhance patient advocacy and include being empathetic, nurturing, ethical, objective, assertive and persistent. Nurse states such as fear, fatigue, frustration and burnout have profound negative impact on patient advocacy. The environment includes all the social, economic and legal elements that influence patient advocacy. Interaction arenas pictorially represent areas of overlap of the categories and portray the interaction of multiple variables in the process and outcome of advocacy actions. This conceptual model is relevant to the study because it describes the nurse traits that influence the performance of patient advocacy which is of interest to the authors. It was thus employed in the study by analyzing the data according to the elements in the nurse category of the model.

Nurses provide care to individuals, the family and sometimes the community and coordinate their services with those of other health care professionals. This co-ordination role puts the nurse in the best position to advocate for the patient [2]. Studies have shown that nurse character traits influence the performance of patient advocacy [5].Their advocacy may be geared towards clients perceived to be neglected, vulnerable, uninformed, intimated, complaining or disinterested in their care. They may also advocate for the protection of clients from the often profit-oriented health system and the paternalist attitude of some health professionals [7–9].

Patient advocacy can be seen as a role for all health professionals but has been adopted by nurses. They believe that their proximity to patients makes them best suited for the role than the other health professionals. Nurses also believe that the performance of this role would enhance their image in the eyes of the public and increase their job satisfaction [10, 11]. It also leads to the promotion of patient rights and wellbeing as well as excellence in nursing care [12].

The literature search however, yielded little information on patient advocacy in the Ghanaian and African contexts. In Ghana, studies show that nurses engage in compassion-based activities like reassuring patients in pain [13]. In other African contexts nurses document their actions including their advocacy actions to ensure patient safety [14]. However some studies in the Ghanaian and broader African contexts show that nurses are often overworked and may lack the energy and time to advocate for patients [13, 15, 16] due to insufficient staff numbers [17]. Poor remuneration and lack of resources at work [15, 18] as well as their low level of participation in decision-making in the hospital [18] can also lead to frustration. They may also lose compassion and find it difficult to advocate for patients who may show ingratitude [19].

This study is therefore necessary to address the paucity of literature in the Ghanaian and African contexts. The aim of this study is to explore the nurse characteristics that influence patient advocacy in a Regional Hospital in Ghana. This aspect of nursing practice has not been systematically explored in the Ghanaian and African contexts. This paper reports one aspect of a wider study on patient advocacy.

## Methods

### Design

The study employed the qualitative exploratory descriptive design to explore the nurse characteristics that influence patient advocacy from the perspectives of practicing nurses [20]. The focus of this design is to gather an in-depth understanding of human behaviour and the reasons that govern such behaviour from the perspective of the participants and relies on narratives from participants to explore issues.

### Setting

The setting for the study was the Effia-Nkwanta Regional Hospital, a government hospital in the Western Region of Ghana that offers both specialist and general medical care. The participants were selected from the theatre/recovery, male medical and children’s wards of the hospital. The participants were selected from three different units to enable the researchers obtain the views of a wider group of nurses in the hospital.

### Sampling and procedures of data collection

Three meetings were organized for prospective participants at the selected units in the hospital to explain the purpose of the study and what the participants would be asked to do. They were also given participants information sheets which contained additional information and the first author’s contact for further enquiries if necessary. They were then allowed to make their own decisions regarding their participation. Those who agreed to take part in the study signed consent forms and were recruited using the inclusion criteria of more than two years working experience and an educational qualification of at least a diploma. Newly qualified nurses who might not have gained enough experience with patient advocacy were excluded from the study. Additionally with the assistance of the ward in-charges participants who could provide in-depth information on the nurse characteristics that influence patient advocacy were selected since they knew them better. Seventeen professional nurses and midwives were then purposively sampled initially from a population of 190. The sample size was however determined when no new finding was generated (saturation). Saturation was achieved with 15 participants.

A semi-structured interview guide was used to collect data from participants. The interview guide was developed based on the objective of the study and the literature review. Open-ended questions were asked to allow participants to express their thoughts and probes were used to follow-up on participants’ responses. Participants were asked questions such as: Why should nurses be the patient advocates? What skills do nurses require to advocate well? What advocacy actions have you performed in the past? The place and timing of the interviews at the hospital were at the convenience of the participants. One-on-one interviews were conducted and participants were given pseudo-names to ensure confidentiality. The venues included isolated locations in the hospital such as empty side wards and hospital library annexes to ensure privacy. The interviews were conducted in English and audio taped.

### Data management and analysis

The audio-taped interviews were transcribed verbatim and checked for accuracy and completeness by listening to the tapes and comparing them to the transcripts. The principles of thematic analysis were applied in analyzing the data which demand that transcripts are coded, organized and integrated according to existing themes and concepts in a systematic and objective fashion [21]. Transcriptions were saved with different colour fonts for the purpose of differentiating responses from different participants. Codes were then written in the left margin of the transcripts close to the corresponding data. Afterwards the codes were organized and integrated according to the themes in the model in a systematic and objective manner. All the authors discussed the themes and sub-themes to ensure that the meanings in participants’ statements were fully captured after the first author had done the initial data analysis.

### Trustworthiness

Strategies adopted to ensure trustworthiness of this study included the use of the same interview guide and also one interviewer conducting all the interviews. An audit trail was kept for other researchers to verify the processes undertaken in this study. A detailed description of the research setting, methodology and background of the sample have been provided to allow for transferability of the findings in similar contexts [22]. In-depth interviews allowed full exploration of the nurse characteristics that influence patient advocacy. Concurrent data analysis ensured that participants’ comments were further explored in subsequent interviews. Also data was later crosschecked with participants after the interviews to verify their responses. Two participants clarified a few of their responses and the changes were made. These approaches ensured validation of findings as the study progressed.

### Ethical considerations

Ethical clearance was obtained from the Noguchi Memorial Institute of Medical Research and the Ghana Health Service Ethical Review Committee. Informed consent was also obtained from each participant. Participants were given the option to withdraw from the study at any time. We ensured that no participant was stressed by conducting the interviews in the participants’ own chosen venues. The identification codes including PA OO1 to PA 015 were assigned to the participants during data collection. Data were kept under lock and key in a drawer in the researcher’s office and a soft copy version was saved in a password protected computer.

## Results

### Demographic characteristics

The participants had a minimum qualification of diploma in nursing or midwifery and with at least three years working experience. There were 14 females and a male. Twelve of them were aged between of 25–35 years, one between 36 and 45 years and two others were above 55 years. One of the participants was a Muslim and the rest were Christians. Two of the nurses were single, 11 were married and two were widowed.

### Nurse characteristics that influence patient advocacy

The themes derived from the study were nurse traits and nurse states. The nurse traits were characteristics that enhanced their role as patient advocates and nurse states were characteristics that hindered their role as patient advocates.

### Nurse traits

This theme describes subthemes such as being empathetic, nurturing, ethical, assertive and persistent. The participants believed that they possessed the traits that made them better patient advocates because they were empathetic and shared in patients’ problems. They were also nurturing and spent more time with patients and identified their problems. They considered themselves more approachable and accessible to patients which allowed patients to confide in them. Additionally they educated patients and helped them to find their way through the health system. Moreover, they often became substitute mothers for children and settled misunderstandings and comforted angry patients. Participants also explained that they saw their role as a moral responsibility and encouraged patient involvement. They also respected patients’ rights and ensured confidentiality in their advocacy. They were of the view that they had the ability to present patients’ problems tactfully and systematically to other team members due to the experience and maturity they had attained. In addition, they persisted and ensured that patients’ problems were solved even when they faced some challenges.

#### Empathetic

Being understanding and sharing in the thoughts and emotions of another person.

A participant spent time with a sad patient to listen to his problems out of empathy.

*“There was a man here who said his wife was also admitted and he didn’t have money. I just sat by him and then he was just pouring out what he was going through”* (PA 009).

#### Nurturing

Being close, caring and protective of patients.

Almost all the participants (14 out of 15) believed that by the nature of their work since they spend long periods of time with patients and provided them with personal care they were more familiar with patients’ problems thus in a better position to advocate for them.

*“We are on the ward with the patients all the time. We hand over patients, write report on them and interact with them so we know whatever is wrong with them …we are the best to stand in for patients”* (PA 009).

*“Sometimes a doctor comes and the patient may say I’m fine. A nurse goes to feed the patient then he says I cannot swallow. As we feed them we find out other things that concern the patients”* (PA 009).

Moreover most participants (11 out of 15) reported that they were more approachable and accessible to patient compared to other health professionals thus better patient advocates.

*“Patients see nurses as very free …nurses are the main people at the hospital they talk to so whatever they need they will ask the nurse*. *They take all the other health care professionals to be doctors so they feel shy to go and ask them questions”* (PA 014).

In nurturing their patients, five participants provided health education and counseled mothers.

*“We educate them on their diet, exercise, on the dos and don’ts and their medications”* (PA 003).

*“We had a mother whose family pressurized her to take her child from the ward to the spiritualist. Then we came in and were able to convince her to stay on the ward while the spiritualist prayed for the baby”* (PA 013).

A few (3 out of 15) also helped patients to find their way through the health system.

*“We don’t do some types of laboratory tests here …they have to go to a private lab. We have some selected places that we do direct them to”* (PA 011).

One participant explained that sometimes nurses also became substitute mothers and took care of children in the absence of their parents.

*“If we realize that there are other children at home and no one could come and stay with the child on the ward we let the child stay in the ward and the nurses take care of the child …the mother goes and comes in the morning”* (PA 013).

A few participants (2 out of 15) settled misunderstandings and comforted angry patients.

*“Patients sometimes have misunderstandings concerning bills and we come in and explain things to bring peace”* (PA 014).

*“Often some staff members insult the patients. We nurses calm the patients down then we later talk to the staff to be patient”* (PA 011).

#### Ethical

Rendering care by following laid down principles and exhibiting high moral standards. Five participants saw their role as patient advocates as a moral responsibility which boosted their job satisfaction.

*“When you are a Christian the Bible says we should help the meek. It feels very good when I know that I helped this patient”* (PA 005).

Three participants encouraged patient involvement, explained procedures and respected patients’ rights.

*“We had a patient with myocardial infarction who always complained that he disliked the way he felt when he took a particular drug. I told him I would tell the doctor but I would also want it to come from him. At first, he didn’t want to so I hammered on it. After he explained it to the doctor, the doctor changed it. I always tell patients that the doctor or nurse is not a soldier to arrest you so ask them. You have the right to know whatever is wrong with you”* (PA 009).

*“If I want to give you treatment and you are refusing I will convince you. Should the patient still refuse the treatment, I would just document it”* (PA 006).

A participant also ensured confidentiality during communication. *“We talk undertone or we isolate them and speak to them. That will make them voice out their problems”* (PA 001).

#### Assertiveness

Acting with confidence and tact and boldly state one‘s opinion about patient care.

Most participants (8 out of 15) stressed the importance of good communication skills in convincing patients and presenting patients’ problems tactfully and systematically to team members in order to get a good response.

*“If you are a patient’s advocate you tell them the reason why you want them to make certain choices. You explain very well and give them scenarios for them to understand. You will have to take your time …the communication should be done well”* (PA 013).

“*You can tell a doctor that I think this and that should be done. You present it tactfully and nicely so that they will listen to you. You have to give tangible reasons”* (PA 015).

Five participants explained that experience and maturity made them more assertive and better patient advocates.

*“It bothers on your character, track record and experience. When they realize you are concerned about your patients, they listen to your submissions”* (PA 015).

*“When you are matured your way of approaching patients is different because maybe you’ve had some past experiences that were bad and you want to really correct them”* (PA 013).

#### Persistent

Continuing steadfastly in an attempt to achieve set goals especially when faced with many challenges.

Some participants (5 out of 15) ensured that patients’ problems were solved despite the challenges they sometimes faced.

*“We are inquisitive and so we tend to probe further about issues. After wardrounds, if we talk to patients we get more information. We ask more questions and we want to know everything and it helps”* (PA 004).

*“We go and pressurize them before they give the drugs at the dispensary. Some of them they are stubborn. They won’t freely do what you want them to do. I think they don’t understand what we mean by emergency”* (PA 011).

### Nurse states

Despite possessing the traits to advocate for patients, heavy workload made them too tired to advocate for patients. The poor conditions of work of nurses and lack of appreciation sometimes from patients were also discouraging. Additionally some felt powerless to influence patient treatment plans. These assertions formed two subthemes under this theme namely fatigue and frustration.

#### Fatigue

A few participants (4 out of 15) in this study explained that heavy workload and the additional duties they usually performed made them too tired to advocate for their patients.

*“The work is overloading us. Sometimes we don’t talk to them very well; we don’t do all these things well”* (PA 005).

*“I’m on the ward as a nurse; I’m also doubling up as a triage team member and as an acting in-service training coordinator. So there are a whole lot of things surrounding me. At the end of the day, I wouldn’t be able to achieve whatever I wanted to do* (sighs)*. Sometimes we become overwhelmed and we don’t inform patients of whatever is happening”* (PA 013).

#### Frustration

Six participants expressed frustration with their poor conditions of work and lack of resources which negatively affected their patient advocacy role.

*“When I’m sick I buy my own drugs and you are telling me to advocate for somebody? There is no way you get a sick person to really do proper patient advocacy. Sometimes the environment we work in is even appalling. If you need something to work with you don’t get it. You have to use your own money to buy it for the patient”* (PA 13).

A third of them (5 out of 15) also felt powerless to influence patient treatment plans.

*“We might say something about a patient’s treatment but the final decision is for the doctor to make but if the nurse had the power and the doctor does not agree we can decide to do it”* (PA 014).

*“Sometimes you advocate for your patient and it’s not accepted. The doctor would tell you there is nothing he can do. Sometimes it is very hurting when someone tells you her problems and you are not able to give the solution. It makes you a little bit* (sighs) *…they think you are not knowledgeable”* (PA 001).

A few of them (2 out of 15) also felt frustrated when patients failed to appreciate their advocacy efforts.

“*Some of the patients don’t appreciate us. Some will not even say thank you. We would be going up and down. Sometimes I use my phone to make calls for patients. So some of us nurses coil up in our shells then we just look at you”* (PA 002).

### Additional findings

The study revealed that the participants were also compassionate and followed up their understanding of patients’ problems with actions to solve them. They pleaded on patients’ behalf to have their needs met and provided material and financial assistance to patients. They also facilitated their care, updated anxious relatives on patients’ condition and allowed them access to patient whenever possible.

#### Compassionate

Understanding another person’s suffering and taking action to relieve it.

Most participants (10 out of 15) identified with and understood patients’ feelings and difficulties thus they communicated their wishes and pleaded on their behalf.

*“Patients are served with two meals; morning and afternoon. When we go for management meetings oftentimes we propose that patients are given meals three times in a day and the meals must be served before they even take their drugs”* (PA 009).

*“Some people are not able to foot their bills so we invite the social workers, we talk to them and plead on their behalf and some are allowed to go home”* (PA 003).

Many participants (9 out of 15) also provided patients with material and financial assistance.

*“We collect unused drugs from discharged patients and keep them for other poor patients who may need it when the time comes at no cost”* (PA 011).

*“We comb around the ward going from one person to the other to get something to buy drugs for the patient”* (PA 012).

Two participants facilitated patient care and acted quickly to save lives.

*“We triage patients so that those who are seriously ill would be given immediate attention”* (PA 004).

*“There was this child whose haemoglobin level was 1.8g/dl and the child was gasping so we had to run quickly to get the blood. In the space of 15 minutes and after 50ml the child was sitting up. I was so happy I was able to save that child”* (PA 013).

About half (7 out of 15) of the participants updated relatives on patient’s their condition and gave them access to visit them.

*“In the theatre the relatives become anxious and so we give them updates on what is happening just to calm them down”* (PA 004).

*“At the recovery ward, we give them emotional support …we allow their close relatives to come in to see them”* (PA 007).

## Discussion

The participants believed that they were empathetic, nurturing, ethical, assertive and persistent; traits which made them better patient advocates than other members of the health team. However, certain states such as fatigue and frustration often interfered with the performance of patient advocacy. These findings are consistent with earlier findings that certain nurse characteristics influence patient advocacy [3–5]. However, just one participant lent a listening ear to a patient who was lamenting about his problems [23]. Due to their nurturing trait the participants performed advocacy actions such as spending more time with patients and rendering personal care which enabled them to witness patients’ problems [10, 24]. Nurses felt that because they were close, more approachable and accessible to the patients, they perceived them to be less threatening than other health professionals [10, 25, 26]. They also educated and counseled patients and relatives [25, 27] and assisted them with navigation of the health system [7, 28]. Again, they cared for babies on the ward in the absence of their caregivers [29], settled misunderstandings between patients and other staff and comforted angry patients [25].

The participants were ethical and perceived the role as a moral responsibility [5], encouraged patient involvement in their care and respected their right to refuse treatment [4, 30]. They also ensured confidentiality during conversation with patients [31]. They were tactful and systematic when communicating patients’ problems to team members [3, 4] qualities they had developed through experience and maturity [5, 9]. Furthermore, the participants ensured that patients’ problems were solved despite facing many challenges. This contradicts a previous assertion that when nurses perceive they do not have the power to influence patients’ treatment plans, they hardly advocate for them [9, 10, 32]. Fatigue resulting from heavy workload and the additional duties some participants undertook however prevented them from advocating for patients as reported by other authors from Australia, South Africa and Ghana [13, 15, 16] a problem often attributed to staff shortage [17]. The poor conditions of work and lack of resources was a source of frustration for participants in the performance of patient advocacy [15, 18]. Their inability to influence important decisions about patient care [18] and the lack of appreciation from patients also discouraged further advocacy [19].

Additional findings from the study indicate that the participants were more compassionate than empathetic because they followed up their understanding of patients’ problems with actions to solve them. In so doing they provided material and financial assistance to patients [31] and acted proactively in emergencies [25]. Some of them also updated anxious relatives on their patient’s condition and sometimes allowed them access to visit critically ill patients [7, 33]. Although not included in the model compassion was revealed to be an important nurse trait in patient advocacy.

### Limitations

The conduct of the study in a Government owned regional hospital means findings may not be reflective of what happens in private, district or teaching hospitals and the use of a purposive sampling method may mean the sample may not be representative of the whole population. Additionally the use of open-ended questions in the semi-structured interview guide produced unique responses that resulted in a wide range of responses.

## Conclusion

The literature on patient advocacy in the Ghanaian and African contexts is scarce although the role is widely practiced especially among nurses. The study has revealed the nurse characteristics that influence the performance of the patient advocacy role which are similar to those identified in USA, Sweden and Iran. Nurse traits such as being empathetic, nurturing and assertive facilitate patient advocacy and nurse states such as fatigue and frustration hamper the performance of the role. However, being compassionate emerged from the study as an additional trait that enhanced patient advocacy. The findings may thus help nurses understand how their characteristics influence their role as patient advocates and help them develop the enhancing traits and overcome the negative states. It may also help in the training, recruitment and mentoring of nurses and enhance multidisciplinary teamwork. The need for further studies to be conducted on patient advocacy in the Ghanaian and African contexts has also been highlighted.
